# Are cervical multifidus muscles active during whiplash and startle? An initial experimental study

**DOI:** 10.1186/1471-2474-9-80

**Published:** 2008-06-05

**Authors:** Gunter P Siegmund, Jean-Sébastien Blouin, Mark G Carpenter, John R Brault, J Timothy Inglis

**Affiliations:** 1 MEA Forensic Engineers & Scientists, Richmond, BC, Canada; 2 School of Human Kinetics, University of British Columbia, Vancouver, BC, Canada; 3 ICORD, University of British Columbia, Vancouver, BC, Canada; 4 MEA Forensic Engineers & Scientists, Lake Forest, CA, USA

## Abstract

**Background:**

The cervical multifidus muscles insert onto the lower cervical facet capsular ligaments and the cervical facet joints are the source of pain in some chronic whiplash patients. Reflex activation of the multifidus muscle during a whiplash exposure could potentially contribute to injuring the facet capsular ligament. Our goal was to determine the onset latency and activation amplitude of the cervical multifidus muscles to a simulated rear-end collision and a loud acoustic stimuli.

**Methods:**

Wire electromyographic (EMG) electrodes were inserted unilaterally into the cervical multifidus muscles of 9 subjects (6M, 3F) at the C4 and C6 levels. Seated subjects were then exposed to a forward acceleration (peak acceleration 1.55 g, speed change 1.8 km/h) and a loud acoustic tone (124 dB, 40 ms, 1 kHz).

**Results:**

Aside from one female, all subjects exhibited multifidus activity after both stimuli (8 subjects at C4, 6 subjects at C6). Neither onset latencies nor EMG amplitude varied with stimulus type or spine level (p > 0.13). Onset latencies and amplitudes varied widely, with EMG activity appearing within 160 ms of stimulus onset (for at least one of the two stimuli) in 7 subjects.

**Conclusion:**

These data indicate that the multifidus muscles of some individuals are active early enough to potentially increase the collision-induced loading of the facet capsular ligaments.

## Background

The cervical facet joints are a source of neck pain in about half of chronic whiplash patients [1]. In addition to guiding better diagnostic and treatment techniques [2,3], this finding provides an anatomical focus to biomechanical studies aimed at understanding the aetiology of whiplash injuries. Pinching of the posterior synovial fold of the cervical facet capsular ligament is one possible injury mechanism [4], but more attention has been devoted toward excess strain of the capsular ligament itself [5-8]. Injurious levels of strain have been observed in some capsular ligaments when loads simulating a rear-end collision were applied in-vitro [7,8]. More recently, allodynia – measured as paw withdrawals in a rat model – has been correlated to levels of capsular ligament strain relevant to whiplash injury [9], and Group III and IV afferents from the facet joint capsule have demonstrated a graded response to mechanical loading in an in-vivo goat model [10].

Anatomically, the cervical facet capsule contains fine, unmyelinated nerves that likely have nociceptive function [11]. Distending these ligaments by injection of contrast media has produced whiplash-like pain patterns in normal individuals [12]. Tendons of the cervical multifidus muscles insert directly onto the capsular ligaments [13,14] and it has been postulated that multifidus activation during the neuromuscular response to a rear-end automobile impact could increase the strain in the capsular ligaments above that imposed passively by the impact-induced head and neck dynamics [7,13]. Prior work has shown early multifidus activation during a whiplash response in one of three subjects [15], however, it remains unclear whether this reflex response will be present in a larger group of subjects.

The neuromuscular response to a whiplash exposure contains both a postural and a startle response [16,17]. This combined postural/startle response was observed in surface electromyograms of the sternocleidomastoid and cervical paraspinal muscles with and without the loud sound of a vehicle crash [16], although muscle activity was larger when the acceleration was accompanied by a loud sound [18]. These prior findings suggest that a startle response – presumably evoked by the crash motion, the noise, or some combination of the two – amplifies the superficial neck muscle response during an unexpected rear-end collision. If either the crash-induced motion or a loud noise also evokes a reflex response in the deep multifidus muscles, then this muscle contraction could add to the loads borne by the facet capsule during a rear-end automobile collision and possibly affect the capsular ligament's injury potential.

Based on this line of reasoning, our broad goal is to establish whether reflex activation of the cervical multifidus muscle contributes to straining the facet capsular ligament and thereby contributes to the genesis of whiplash injury. In this study, we examine one step in this broader goal and specifically address whether a reflex contraction of the multifidus muscle is evoked by either the postural response (a seated horizontal acceleration without the noise of impact) or a startle response (a loud noise without a postural perturbation). If either of these stimuli generates a reflex response in multifidus, our broader goal warrants continued investigation; if neither stimulus generates a reflex response, then we can conclude that multifidus likely does not play a role in facet capsule injury in whiplash.

## Methods

### Subjects and consent

Nine subjects (6M, 3F) participated in the experiment. Male subjects were 30 ± 6 years old, 177 ± 6 cm tall and weighed 79 ± 5 kg; female subjects were 30 ± 1 years old, 166 ± 5 cm tall and weighed 68 ± 6 kg. None of the subjects had a history of whiplash injury, medical conditions that impaired sensory or motor function, or prolonged neck or back pain during the preceding 2 years. Subjects did not ingest caffeine or nicotine for two hours before the experiment. All subjects gave written inform consent and the experiment was approved by the UBC Clinical Research Ethics Board and conformed to the Declaration of Helsinki.

### Instrumentation

**Figure 1:**
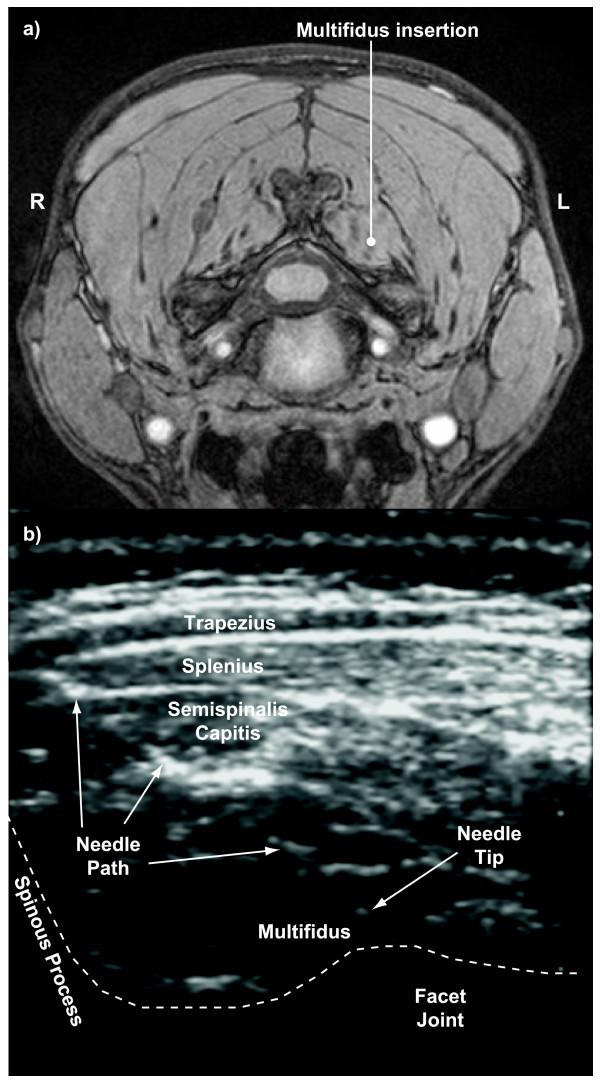
**MRI and ultrasound images showing wire insertion**. a) axial slice of a magnetic resonance scan at C4 level showing desired insertion path to the multifidus muscle and b) ultrasound image showing actual needle path and tip during insertion into the multifidus muscle.

EMG activity of the left multifidus muscles was measured using twisted pairs of insulated 0.05 mm wire (Stablohm 800A, California Fine Wire, Grover Beach, CA) with 1–2 mm of wire exposed at each recording tip. The recording tip of each wire was hooked to anchor it in the muscle tissue. After first identifying multifidus and any major vessels on each subject's magnetic resonance (MR) scan (Phillips Gyroscan Intera 3.0T), wires were inserted into the multifidus muscles at the C4 and C6 levels using 25 gauge needles under ultrasound guidance (Sonos 5500, Agilent Technologies, Andover, MA) (Figure 1). Ultrasound was again used during wire extraction, and although the wires were harder to visualize during extraction (without the needle present), tissue displacement at the hooked end was readily apparent during wire extraction. The recording ends of all wires were in the same location before and after the experiment, which meant that the wire has not moved substantively relative to the surrounding muscle tissue during the exposures. EMG signals were amplified and band-pass filtered (30–1000 Hz) using a Neurolog system (Digitimer, Welwyn Garden City, Hertfordshire, England). Head acceleration was measured with a nine accelerometer array (Kistler 8302B20S1, Amherst, NY) arranged in a 3-2-2-2 configuration [19] and sled acceleration was measured with a uniaxial accelerometer (Sensotec JTF3629-05, Columbus, OH). Head and torso displacements were measured with a motion analysis system (Pheonix VZ4000, Burnaby, BC). Transducer signals were low-pass filtered (1000 Hz) and, together with the EMG signals, simultaneously sampled at 2 kHz. Displacement data were acquired at 100 Hz per marker.

### Test procedures

**Figure 2:**
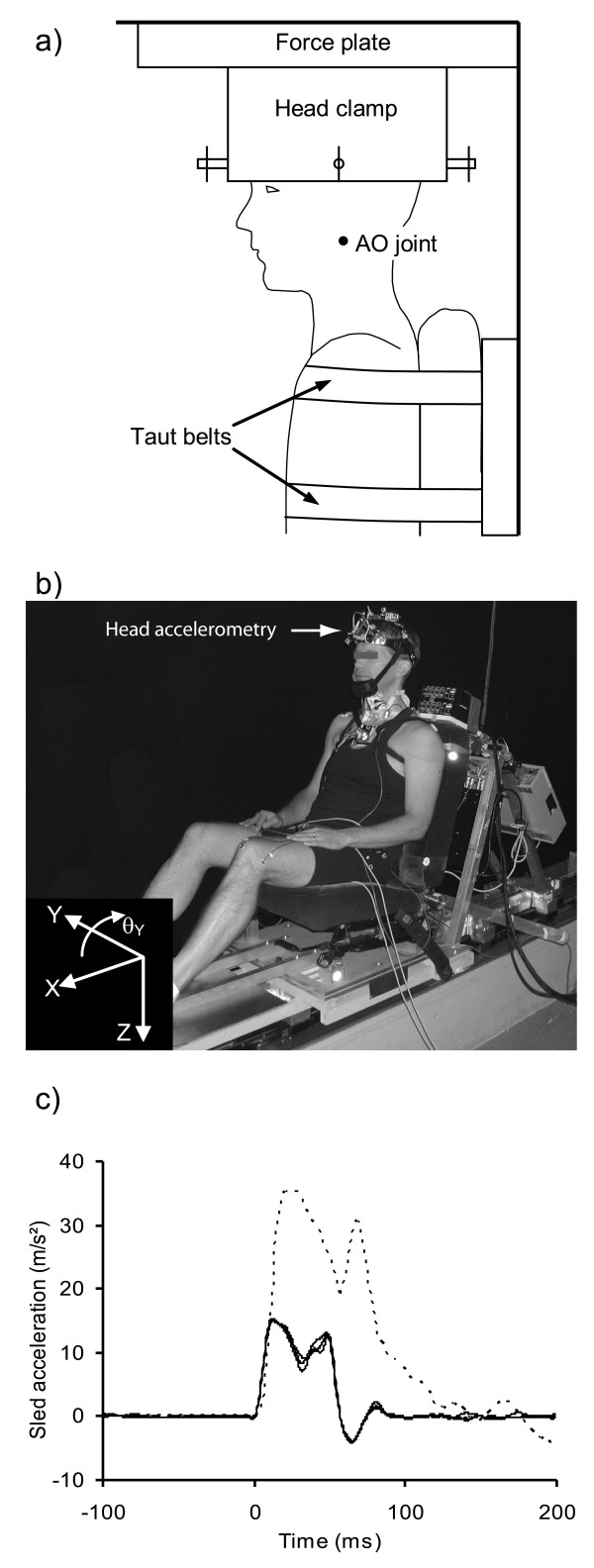
**Experimental setup**. Schematic and photograph showing a) the head clamp and force plate used for the isometric contractions, b) the sled configuration, and c) the sled acceleration pulses. Three superimposed pulses are shown to illustrate the repeatability of the pulse. The dashed line shows a vehicle-to-vehicle collision pulse with a speed change of 8 km/h recorded during earlier experiments [19].

Subjects first performed 5-second isometric maximal voluntary contractions (MVCs) from the neutral position in eight directions (flexion, extension, left and right lateral flexion, and the 45° points between these 4 primary directions) to provide normalizing data for the dynamic EMG recordings. For the MVCs, a subject's head was firmly clamped to an inverted force plate (Bertec 4060H, Worthington, OH) and their torso firmly strapped to a rigid seat back (not the same seat used for the exposures described below) (Figure 2a). For their exposure to the acceleration and acoustic stimuli, subjects were seated in an automobile seat (1991 Honda Accord front passenger with the head restraint removed) mounted to a feedback-controlled linear sled (Figure 2b). The sled consisted of two linear induction motors (IC55-100A7; Kollmorgen, Kommack, NY) mounted through linear bearings to 6 m horizontal rails rigidly fastened to a concrete base. Sled motion was programmed through a 500 Hz position-feedback controller and was thus insensitive to subject mass. Subjects were instructed to adopt a comfortable seated posture, face forward, rest their forearms on their lap, and relax their face and neck muscles. Seated subjects were first exposed to a single unexpected loud acoustic stimulus (124dB, 1000 Hz, 40 ms duration) capable of evoking a startle reflex [20]. After a rest period of at least 3 minutes, subjects experienced a single forward horizontal acceleration pulse (a_peak _= 1.55 ± 0.02 g; t_peak _= 16 ± 1 ms; Δt = 59 ± 1 ms; Δv = 0.50 ± 0.01 m/s, noise<82 db, Figure 2c). After the pulse, the sled traveled at 0.50 m/s for 500 ms (beyond our period of interest) before decelerating linearly to rest at 0.05 g. A single exposure to each stimulus was used because multiple exposures could be confounded by the rapid habituation observed to loud acoustic stimuli [21,22] and seated perturbations [23,24].

### Data reduction

All EMG data were high-pass filtered (50 Hz) to remove motion artifact present in some tests. The onset of EMG activity was determined using a log-likelihood-ratio algorithm [25,26] and then confirmed visually. For both the acceleration and acoustic stimuli, the root-mean-squared (RMS) amplitude of the EMG signals was computed using a moving 20 ms window. These RMS values were then normalized by the maximum RMS EMG (also using a 20 ms window) observed during the MVC contractions.

For the acceleration and acoustic stimuli, three kinematic parameters were calculated: i) the horizontal acceleration of the head's centre of mass in the lab frame (a_x_), ii) the horizontal displacement (retraction, r_x_) of the atlanto-occipital (AO) joint with respect to the centre of the T1 vertebral body, and iii) the extension angle (θ_y_) of the head in the lab frame. The center of the T1 vertebral body relative to the manubrium and C7 spinous process was determined from each subject's pre-test MR scan. All time-varying kinematic signals were set to zero at the start of the stimulus.

### Statistical analysis

All dependent variables were first tested for normality using a Shapiro-Wilks test. Differences between the stimuli (acceleration/acoustic) and spine level (C4/C6) were tested using a two-way repeated-measures ANOVA and Tukey post-hoc tests for normally distributed variables. For variables not normally distributed, a non-parametric Friedman ANOVA and post-hoc Wilcoxon matched pairs tests were used. All tests were performed using Statistica (v.6.1, Statsoft, Tulsa, OK) with a significance level set at p < 0.05.

## Results

**Figure 3:**
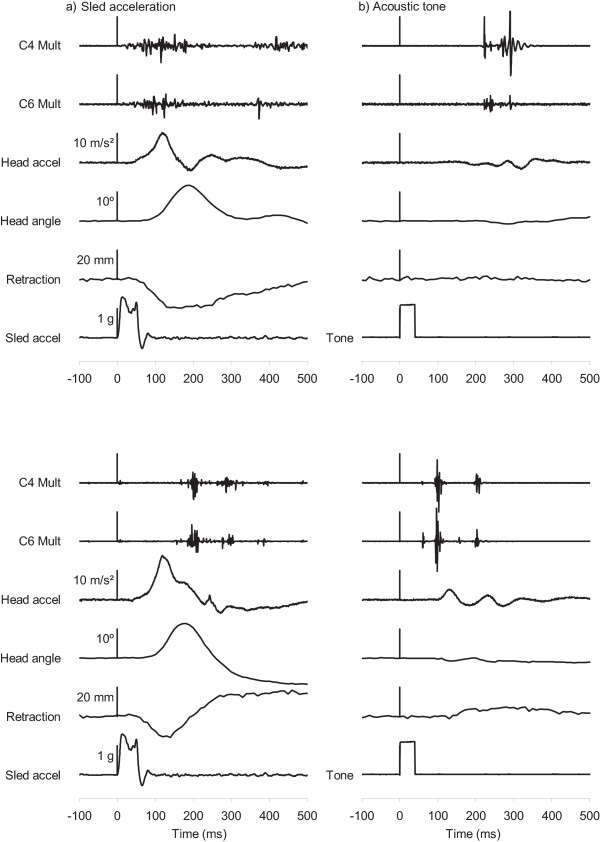
**Exemplar data**. Exemplar data for two subjects showing the raw electromyographic activity and kinematics parameters observed during a) the sled perturbation and b) the loud acoustic tone. The vertical scale bars are aligned with time t = 0 ms. EMG are presented in arbitrary units that are the same for both stimuli.

Eight of the nine subjects exhibited multifidus activity after both stimuli, as demonstrated by the exemplar data of two subjects in Figure 3. The remaining subject exhibited no multifidus activity to either stimulus – despite clear multifidus activity during her MVC – and was excluded from the statistical analysis of the EMG data. Multifidus activity was present in all eight subjects at the C4 level and in six subjects at the C6 level. Supramaximal muscle activity was observed either at one level or to one of the two stimuli in six subjects.

There were no differences in the muscle response amplitude between the acceleration and acoustic stimuli or between the two spine levels (p = 0.32) (Table 1). There were also no differences in the onset latencies between either stimulus types or recording levels (p = 0.41). There was however considerable variation in the onset latencies between subjects, with multifidus onset occurring within 160 ms of stimulus onset in five subjects during the sled acceleration and six subjects (four of which were common) during the acoustic stimulus.

**Table 1:** Mean ± SD and median (range) of muscle onset times, normalized muscle amplitudes, kinematic amplitudes and time of peak kinematics.

	Sled	Startle
EMG onset time (ms)		
C4 level	129 ± 60	123 ± 64
	140 (34 – 191)	105 (58 – 222)
C6 level	232 ± 175	182 ± 120
	194 (66 – 571)	126 (60 – 417)
EMG amplitude (%MVC)		
C4 level	137 ± 168 ^a^	136 ± 123 ^a^
	107 (12 – 532)	80 (39 – 337)
C6 level	40 ± 25	57 ± 53 ^a^
	36 (9 – 77)	28 (15 – 150)
Kinematics		
Head accel (m/s^2^)	9.7 ± 3.3	1.9 ± 1.0
	10.1 (5.6 – 15.2)	1.6 (0.5 – 3.5)
Head extension (°)	15.6 ± 5.6	1.9 ± 3.1 ^a^
	12.6 (10.0 – 25.9)	0.5 (0.1 – 8.8)
Retraction (mm)	18.4 ± 6.9	4.3 ± 2.4 ^a^
	19.4 (8.7 – 31.3)	5.2 (1.0 – 6.6)
Time to peak kinematics (ms)		
Head accel	123 ± 11	272 ± 168 ^a^
	122 (106 – 145)	215 (124 – 581)
Head extension	208 ± 51 ^a^	557 ± 200
	190 (170 – 330)	510 (290 – 770)
Retraction	173 ± 48	373 ± 187
	160 (110 – 240)	330 (130 – 710)

^a ^non-normally distributed, Shapiro-Wilks p < 0.05

All subjects exhibited a well-defined forward head acceleration, head extension and retraction of the AO joint relative to T1 when exposed to the sled acceleration (Figure 3). The peak kinematic responses for the acoustic stimulus were smaller (p < 0.02; Table 1) and also varied in direction, with the initial head acceleration forward in four subjects and rearward in the other subjects. Kinematic peaks occurred earlier and were less variable for the sled acceleration compared to the acoustic stimulus (p < 0.03; Table 1).

## Discussion

Based on the data presented here, the cervical multifidus muscles of some individuals are active during either postural or startle responses. Of our nine subjects, eight subjects exhibited multifidus activity following the stimuli and seven subjects responded within 160 ms to at least one of the two stimuli. As a result, this study establishes that multifidus could play a role in straining the capsular ligament of a large segment of the population during a rear-end collision. Of course the single subject in the current study who responded to neither stimulus may represent a segment of the population whose multifidus muscles do not react to these stimuli, and this subject should not be dismissed outright as atypical given the low number of subjects we tested. Despite this caveat, these findings lend support to the proposition that multifidus activation may play a role in the genesis of some whiplash injuries, although more work is needed before we can conclude whether it actually contributes to injuring the facet capsule ligament.

To be relevant to whiplash injury, the multifidus muscle must activate both early and forcefully during its response to a collision. Peak capsular ligament strain occurs about 200 ms after the onset of T1 acceleration [8], and T1 acceleration begins about 25 to 35 ms after vehicle impact [19]. Assuming meaningful levels of muscle force are generated 75 to 100 ms following activation [27,28], multifidus activation must occur within 125 to 160 ms after impact (i.e., sled acceleration onset) to coincide with the collision-induced peak in capsular ligament strain. Of our nine subjects, multifidus was active within 125 ms in 4 subjects following the sled acceleration (see example in upper panel of Figure 3a) and 6 subjects following the startling tone (see example in lower panel of Figure 3b). In one additional subject, multifidus was active within 160 ms of the sled acceleration onset. Thus, the multifidus muscles responded to at least one stimulus sufficiently early to potentially contribute to a facet capsular injury in slightly more than half our subjects. For individuals startled by an unexpected rear-end impact, it may not matter whether multifidus activation was mediated by a startle or postural response: activation by either stimulus before peak retraction could contribute to increasing facet capsule strain. For individuals whose multifidus muscles do not respond to either stimulus, or for individuals who have both delayed multifidus activation to a sudden acceleration and no startle response, the multifidus muscles may not affect capsule strain and therefore play no role in generating a whiplash injury.

The force generated by the multifidus muscle in our subjects was not measured, but about half our subjects exceeded their MVC level during either the acceleration or acoustic stimuli. Supramaximal muscle activity has been observed by others studying whiplash [29], but it remains unclear why or how this occurs. Subjects may not have exerted maximal efforts during their MVCs; however, the neck moments measured in our male subjects (flexion 12 ± 6 Nm; extension 20 ± 8 Nm) are similar to maximal neck moments (flexion 13 ± 3 Nm; extension 24 ± 7 Nm) measured by others [30]. It is also possible that multifidus may not recruit fully during contractions designed to maximize the horizontal force at the forehead or may be recruited differently in the seats used for the MVC and the acceleration/acoustic exposures. Alternatively, short, reflex muscle activations may generate more synchronous bursts of action potentials than do 5 second maximal voluntary efforts, and summation of these synchronous action potentials yields a greater EMG signal during transient reflex activations. Further work is needed to determine why this supramaximal activity occurs and how to estimate multifidus muscle force from these data.

Although we did not measure muscle force, the maximum force that the multifidus muscle can generated can be estimated from data in the literature. The area of multifidus insertion onto the facet capsule varies between 9 and 96 mm^2 ^(average of 48 ± 22 mm^2^) [6], which for an isometric tetanic stress of 0.44 MPa [31], corresponds to an applied force of 9 to 42 N (average 21 ± 10 N). During elongation, force increases by a factor of 1.2 to 1.6 depending on lengthening velocity [32] and therefore the maximum force that could be applied to the capsule is between 11 and 67 N (average 29 ± 14 N; a factor of 1.4 is assumed for this average and SD). Prior estimates of the peak multifidus force (42 to 55 N) using mathematical models of the male neck are greater than this average [33,34], but slightly less than the maximum value computed here.

The loads applied by a maximally-active multifidus muscle to the facet capsule are potentially a large proportion (about 64% on average) of the quasi-static loads (21 to 93 N; 45 ± 21 N) required to cause sub-catastrophic failures in the capsular ligament [7]. Sub-catastrophic failure loads under dynamic conditions have not been reported, but increasing catastrophic failure loads have been observed with increasing elongation rates (Table 2). Peak ligament elongation rates of about 50 mm/s have been estimated for whiplash loading with an average T1 acceleration of 2.3 g over 100 ms [see Figure 5 in reference [35]]. This level of T1 acceleration is consistent with a vehicle speed change of about 8 km/h [19], and higher strain rates likely occur in more severe collisions. If we assume that catastrophic and sub-catastrophic failure loads scale similarly, then the data in Table 2 suggest that sub-catastrophic failure loads will be up to 1.5 times higher under dynamic conditions with elongation rates of up to 100 mm/s than under quasi-static conditions. This assumption suggests the dynamic loads required to cause sub-catastrophic failures in the capsular ligament would be in the region of 68 ± 32 N at elongation rates of 100 mm/s. Thus even under dynamic conditions, the loads applied by a maximally-active multifidus muscle to the facet capsular ligament are potentially large (43% on average) compared to the capsule's estimated sub-catastrophic failure loads. During severe collisions inducing capsule elongation rates greater than 100 mm/s, the sub-catastrophic failure loads will be higher and the proportional contribution of multifidus activation to capsule failure will be lower. Joints with large multifidus/capsular insertion areas, low partial-rupture loads and high levels of multifidus activation during the collision might be particularly prone to injury. Unfortunately, the large within-individual variations in insertion area [6] and partial-rupture load [7], combined with the large between-subjects variability in multifidus activation timing and amplitude observed here, make it difficult to predict who is a risk for this type of injury.

**Table 2:** Catastrophic failure loads of isolated facet capsular ligaments as a function of elongation rate.

Study	N Gender	Elongation rate (mm/s)	Failure load (N)
Winkelstein et al. (1999) [43]	12M	0.0083	84 ± 37
Siegmund et al. (2001) [7]	13F	0.0083	94 ± 31
Myklebust et al. (1988) [44]	2–5*	10	108 ± 40**
Winkelstein et al. (1999) [43]	12M	100	136 ± 60
Ivancic et al. (2007) [45]	32	723	220 ± 84
Shim et al. (2006) [46]	15M	9600 – 13,600	260 ± 112

* N at each cervical level from C2/3 to C7/T1 between 2 and 5

** average of the means and standard deviations at cervical level from C2/3 to C7/T1; also values reported here are half those reported by Myklebust et al (1988) because left and right ligaments were tested simultaneously.

The relatively large force that multifidus can apply through the capsular ligament combined with the large activation levels occasionally observed during a pure startle raises the question of why facet capsule injuries do not occur – at least in some individuals – when startled. We do not know the answer to this question, but it may be that the capsular ligaments and multifidus muscles of a particular individual co-develop and are well matched for normal neck movements and startle responses in normal or near-normal postures. Whiplash-induced neck motion may pre-strain the capsular ligament and make it more vulnerable to multifidus-induced loading – or vice versa. Further work is needed to explore this question.

The high percentage of Type I muscle fibres [36], the small number of vertebra spanned [14] and the low moment generating capacity [14] of the cervical multifidus muscles may suggest this muscle has a primarily postural role. Recent evidence, however, suggests the neck multifidus muscles exhibit phasic activity during isometric contractions and voluntary head movements in addition to receiving neural signals common to the superficial neck muscles [15,16]. Thus posture-related activity – and perhaps more importantly movement-related activity – in the multifidus muscle could conceivably provoke or exacerbate pain in an injured capsular ligament. Moreover, the high number of muscle spindles in multifidus [37] suggests an important role for multifidus in directly sensing spine joint position and perhaps contributing to head position sense. If pain from an injured capsular ligament inhibits or alters tonic or phasic multifidus activity, then altered somatosensory information from muscle spindles could also contribute to the dizziness experienced by many whiplash patients [38]. Indeed, increased joint position error in whiplash patients has been previously attributed to mechanoreceptor dysfunction in the cervical spine [39,40], and a dysfunctional interaction of the capsular ligament and the multifidus muscle could explain these symptoms.

For this experiment, we used a sled acceleration that was less severe and an acoustic stimulus that was more intense than those experienced by many whiplash-injured patients. Although our acceleration pulse yielded a low overall speed change, its leading edge matched the onset of a collision with a speed change of 8 km/h (Figure 2c) [41], and as a result, the onset times we observed likely apply to more severe collisions. The amplitude of surface EMG from superficial neck muscles increases with both increasing sled acceleration and velocity change [42], and based on this pattern, we would normally expect larger multifidus EMG activity in higher severity collisions. However, since supra-maximal EMG activity was already evoked in some subjects by our low-severity sled perturbation, it is not clear whether there is scope for increased multifidus EMG with increased perturbation severity. Further work is needed to explore this issue. The acoustic stimulus (124dB) was louder than generated by most vehicle-to-vehicle collisions (92 to 110 dB, unpublished measurements). In this experiment, however, we did not attempt to replicate the noise of a collision; rather we were interested in whether a pure startle response involves multifidus muscle activation. Our loud tone thus served to evoke a startle response without the confounding effect of a postural response, and thereby allowed us to see whether startle – by itself – activated the multifidus muscle. We also did not randomize the presentation order of the startle and perturbation stimuli, and as a result the perturbation response may have been attenuated by the preceding startle stimulus. In an actual rear-end collision, the startle component of the response [16] would likely be evoked by a combination of tactile (interaction with the seat) and acoustic (crash noise) sensory stimulation. Moreover, vehicle occupants who are unexpectedly struck from behind may experience a superposition of the two neuromuscular responses we observed here.

Our subjects were seated in an unmodified automobile seat similar in construction but perhaps different in mechanical properties from more recent automobile seats. Compared to our seat, some modern seats, particularly those with integrated seat belts, likely have a higher seatback stiffness that could produce higher occupant accelerations that in turn could increase the neck muscle response amplitude. Other modern seats, such as some anti-whiplash seats, may have lower seatback restitution values than the seat we used, and thus yield lower occupant speed changes that in turn could decrease the neck muscle response. Further work is needed to explore the effect of the different design trends in modern seats on the timing and amplitude of the neck muscle response.

The ability to generalize our results to the broader population is limited by the small number of subjects we tested. The goal of this experiment, however, was to establish the plausibility of a potential role for multifidus in the genesis of whiplash injury, and given the invasive nature of our experiment, nine subjects was deemed sufficient to establish this plausibility. Based on our results, additional work is now justified using a larger number of subjects and a wider range of impact severities to further explore the role of multifidus in whiplash injury.

## Conclusion

In summary, cervical multifidus muscle activity is evoked by both horizontal sled accelerations and acoustically startling tones. This finding represents one step towards understanding whether reflex activation of the multifidus muscle can exacerbate a whiplash injury involving the cervical facet capsule ligament.

## Competing interests

Authors GPS and JRB hold shares in and are employed by a forensic consulting company that may benefit from publication of these data. Authors JSB, MGC and JTI declare no conflicts.

## Authors' contributions

All authors participated in conceiving and designing the study. Pilot work was carried out by GPS, J–SB, JRB and JTI. GPS and J–SB conducted the experiments with assistance from MGC and JTI. GPS and J–SB analyzed the data with input from the other authors. GPS drafted the manuscript with feedback from all authors. All authors read and approved the final manuscript.

## Pre-publication history

The pre-publication history for this paper can be accessed here:


